# Preferences for HIV pre‐exposure prophylaxis among men who have sex with men and trans women in 15 countries and territories in Asia and Australia: a discrete choice experiment

**DOI:** 10.1002/jia2.70025

**Published:** 2025-08-20

**Authors:** Warittha Tieosapjaroen, Benjamin R. Bavinton, Heather‐Marie A. Schmidt, Curtis Chan, Kim E. Green, Nittaya Phanuphak, Midnight Poonkasetwattana, Nicky S. Suwandi, Doug Fraser, Hua Boonyapisomparn, Michael Cassell, Lei Zhang, Weiming Tang, Jason J. Ong

**Affiliations:** ^1^ The School of Translational Medicine Monash University Melbourne Victoria Australia; ^2^ Melbourne Sexual Health Centre, Alfred Health Carlton Victoria Australia; ^3^ Kirby Institute, University of New South Wales Sydney New South Wales Australia; ^4^ UNAIDS Regional Office for Asia and the Pacific Bangkok Thailand; ^5^ Global HIV, Hepatitis and STIs Programme, World Health Organization Geneva Switzerland; ^6^ The Program for Appropriate Technology in Health Hanoi Vietnam; ^7^ Institute of HIV Research and Innovation Bangkok Thailand; ^8^ Asia Pacific Coalition on Male Sexual Health Bangkok Thailand; ^9^ Asian Pacific Transgender Network Bangkok Thailand; ^10^ Family Health International 360 Hanoi Vietnam; ^11^ The University of North Carolina at Chapel Hill Project‐China Guangzhou China; ^12^ Faculty of Infectious and Tropical Diseases London School of Hygiene and Tropical Medicine London UK

**Keywords:** HIV acquisitions, HIV prevention, men who have sex with men, pre‐exposure prophylaxis, sexual and gender minorities, transgender persons

## Abstract

**Introduction:**

Scaling up pre‐exposure prophylaxis (PrEP) for HIV among men who have sex with men (MSM) and transgender women (TGW) in the Asia‐Pacific region has been slow. We identified the drivers of PrEP use and forecasted PrEP uptake given different PrEP programmes for MSM and TGW living in 15 countries and territories in Asia and Australia.

**Methods:**

Separate online discrete choice experiment surveys for MSM and TGW were distributed in 15 Asian countries and territories and Australia between May and November 2022. We used random parameters logit models to estimate the relative importance of service attributes and predicted PrEP uptake for different programme configurations.

**Results:**

Among 21,943 participants included in the MSM survey and 1522 in the TGW survey, the mean age was 31.7 (±9.5) years and 28.1 (±7.0) years, respectively. Cost emerged as the primary driver of PrEP use for MSM and TGW across countries, followed by the type of PrEP. When switching from the least preferred PrEP programme (i.e. very high service fee, PrEP implant, rare kidney problems as side effects of PrEP and a 2‐monthly clinic visit) to an optimal programme (i.e. free access to PrEP via peer‐led community clinics which offered sexually transmitted infection [STI] testing, and a 6–12 monthly visit), the predicted PrEP uptake could improve by over 50% for MSM in Australia, China, Hong Kong SAR China, Japan, the Philippines, Taiwan (China) and Thailand, and 37% for TGW. Compared to those at lower risk of HIV, free access was more preferred by MSM at a higher risk of HIV, while telehealth was more preferred by TGW at a substantial risk of HIV.

**Conclusions:**

Tailoring services to local contexts, including ensuring affordability, preferred type of PrEP and providing differentiated services, could accelerate the uptake of PrEP among MSM and TGW in Asia and Australia. Novel innovations, such as STI and HIV self‐testing, should be explored as alternatives to conventional testing, given that most MSM and TGW prefer less frequent clinic visits and long‐acting PrEP options.

## 1 INTRODUCTION

To end AIDS as a public health threat by 2030, the Joint United Nations Programme on HIV and AIDS (UNAIDS) aims to reduce global new HIV acquisitions to under 200,000 annually [1]. However, the Asia‐Pacific alone witnessed approximately 260,000 new cases in 2021 [2]. HIV cases are rising among young men who have sex with men (MSM) and transgender women (TGW), notably in Malaysia and the Philippines; two of the fastest‐growing epidemics in 2022 [3]. A possible contributor to this surge is insufficient coverage of biomedical HIV prevention [4].

Pre‐exposure prophylaxis (PrEP) is a safe and highly effective HIV prevention method [5, 6]. Within the Asia‐Pacific region, PrEP was first introduced in Thailand in 2014 [7], and subsidised PrEP was first offered in Australia in 2018 [8] to scale‐up PrEP among MSM [9], leading to a significant reduction of HIV incidence at a population level [9]. However, financial constraints compounded by the pervasive stigma surrounding sex, HIV and/or key populations in the region have impeded the availability and accessibility of PrEP. While numerous nations have initiated PrEP programmes, scale‐up remains limited, with only four countries (i.e. Australia, the Philippines, Thailand and Vietnam) reporting more than 10,000 PrEP initiations in 2022 [10]. Meanwhile, most countries, such as Singapore, India and China, have small‐scale programmes despite substantial populations that could benefit from PrEP [10].

Enhancing the region's efforts to reduce HIV transmission must include PrEP in the national HIV response of each country, along with addressing key populations’ needs to increase equitable PrEP access and uptake. The World Health Organization (WHO) advocates person‐centred PrEP programmes for policy development to prioritise affected community engagement over a top‐down approach to ensure health systems are supportive and responsive [11, 12]. To do so, data concerning key populations’ preferences are required to inform evidence‐based policies and optimize programmes to reach those who would benefit most from PrEP [12].

A discrete choice experiment (DCE) quantifies preferences, particularly when exploring preferences for potential services. Respondents are presented with options describing combinations of various service attributes and then asked to select the option that provides them with the highest satisfaction or utility [13]. The resultant choice data allows analysts to evaluate respondents’ trade‐offs between service attributes and levels. DCEs are a powerful method to predict choices in real‐world settings, with strong external validity [14]. DCEs have been increasingly used in HIV research, such as exploring antiretroviral side effects for people with HIV [15] and key populations’ preferences for PrEP [16]. In this DCE study, we identified which attributes were the most important drivers of choice to use PrEP, evaluated the least and most preferred programmes, and explored the impact of different PrEP programme configurations on the uptake of PrEP among MSM and TGW living in 15 countries and territories in Asia and Australia.

## 2 METHODS

This study was approved by the University of New South Wales (UNSW) Human Research Ethics Committee (HC210729). The WHO Ethics Review Committee exempted the study from review, negating the need for separate ethics approvals in participating countries. Family Health International (FHI) 360 agreed that the UNSW Human Review Ethics Committee could act as the institutional review board. Informed consent was obtained before participants entered the survey. Respondents who completed the survey could enter a prize draw, with the prize values set at 3.5% of each country's median monthly income for both MSM and TGW groups. One participant per country survey was randomly selected to receive the prize. These were delivered via email using voucher codes for retailers available in each country.

### 2.1 Study setting and participants

Between May and November 2022, two separate online cross‐sectional surveys were distributed: (1) the survey for MSM via dating apps (e.g. Grindr and Scruff), social media platforms (e.g. WeChat and Facebook) and local and regional MSM community networks via social media and mailing lists; and (2) the survey for TGW via social media influencers and local and regional TGW community networks via social media and mailing lists. APCOM, a non‐profit organization working on HIV and sexual health issues for MSM in 35 countries in Asia and the Pacific, customized the advertising material to fit the specific context of each country. Further information on our recruitment was published elsewhere [17]. We included MSM and TGW aged 18 years and above, reporting no prior HIV diagnosis and living in the participating high‐income (HIC) (only MSM) or middle‐income (MIC) Asian countries and territories (MSM and TGW), or Australia (only MSM). MICs included Cambodia, China, India, Indonesia, Laos, Malaysia, Myanmar, Nepal, the Philippines, Thailand and Vietnam. HIC included China Special Administrative Region (SAR) Hong Kong, Taiwan (China), Japan and Singapore. MSM self‐identified as gay, bisexual or other men who have sex with men. TGW self‐identified as transgender women. No statistical methods were used to pre‐determine sample sizes, but the minimal sample size needed for each country, based on Orme's rule of thumb, was 167 [18]. Our sample sizes exceeded the minimum requirement and were larger than those reported in previous publications [19, 20].

### 2.2 Survey instrument

Anonymous online surveys were created in Qualtrics (Qualtrics, Provo, UT), professionally translated into 15 local languages, and reviewed by community members. Translations were improved based on suggestions from community members. The surveys asked about (1) demographics, (2) PrEP awareness and use, (3) PrEP preferences (DCE), and (4) social and sexual behaviours. The DCE section featured six choice sets. Each choice set had two PrEP programmes, comprising six attributes with different levels. Respondents were asked to select their preferred programme or an opt‐out option if neither programme was preferred. An example of the DCE question can be found in Appendix S1.

### 2.3 DCE design and attribute selection

Attributes and attribute levels were determined by a literature review and consultations with experts in HIV and DCEs. The ranking exercise was used to rank potential attributes and determine appropriate attribute levels. In the final DCE survey, there were six attributes. Each had 3−6 attribute levels. The final attributes included: (1) type of PrEP (daily oral, on‐demand oral, injectable, long‐acting oral, implant); (2) location to access PrEP (hospital, sexually transmitted infection [STI] clinic, general practice, community clinic run by MSM or TGW, telehealth, pharmacy); (3) cost (free and three additional levels [i.e. low, high and very high] using the currency of the country); (4) side effects (none, interactions with other medications, mild, rare chance of kidney problems, mild pain from injection); (5) visit frequency (every 2, 3, 6 and 12 months); and (6) extra services (no additional service, STI testing, mental health counselling and hormone prescribed [TGW only]). The final attributes and levels are shown in Table 1. A D‐efficient experimental design was created in Ngene software (ChoiceMetrics). This design maximized the information gained from a limited number of participant responses and optimized the precision and reliability of parameter estimates [21]. This meant instead of showing all possible combinations of service configurations, the D‐efficient design randomized the attribute and attribute levels and selected a small number of combinations that provided the most insight into participants’ preferences. Randomization occurs in the presentation of attribute combinations within choice tasks, not in assigning participants to conditions. This approach ensures robust parameter estimates while minimizing the cognitive burden on participants. Additionally, the experimental design contained constraints to display plausible combinations (e.g. PrEP frequency of visit every 2 months or side effects of injection pain will only appear if the type of PrEP was injectable PrEP within the choice set).

**Table 1:** Attributes and attribute levels included

Attributes	Attribute level
Type of PrEP	1. Daily oral PrEP
	2. On‐demand oral PrEP
	3. Injectable PrEP
	4. Long‐acting oral PrEP
	5. Implant PrEP
Service location	1. Hospital
	2. Sexually transmitted infection clinic
	3. General practice
	4. Community clinic run by MSM or TGW
	5. Telehealth
Cost	1. Free
	2. Low cost
	3. High cost
	4. Very high cost
Side effects	1. None
	2. Interactions with other medications
	3. Mild
	4. Rare chance of kidney problems
	5. Mild pain from injection
Visit frequency	1. Every 2 months
	2. Every 3 months
	3. Every 6 months
	4. Once a year

Abbreviations: MSM, men who have sex with men; PrEP, pre‐exposure prophylaxis; TGW, transgender women.

### 2.4 Data analysis

We included respondents who answered at least one DCE question in the analyses. The respondents’ demographic characteristics were presented using descriptive statistics. Random parameters logit (RPL) models with 1000 Halton draws were used to estimate the relative importance of attributes for each country/territory [22]. RPL allows us to capture this variability, providing a more accurate picture of what different groups value. All attribute levels were effects coded [23]. All parameters were initially assumed to follow a normal distribution. Some parameters were set as non‐random parameters to achieve a better model fit according to the Akaike information criteria and log‐likelihood function. A simulation model using RPL was used to predict PrEP uptake, comparing “the least preferred PrEP programme” (including attribute levels least wanted), “the optimal PrEP programme” (including attribute levels most wanted) and “status quo” (including attribute levels currently offered). PrEP uptake percentage is the probability of people accepting PrEP when offered a specific PrEP service programme.

RPL with interaction models were used to explore the heterogeneity of preferences of those who would most benefit from PrEP (i.e. those at substantial risk of HIV acquisition and naïve‐PrEP users). We defined substantial risk as individuals who, in the last 6 months, were either involved in sex work, engaged in condomless anal sex, used drugs before or during sex (i.e. chemsex), had multiple sexual partners, shared injecting equipment, were diagnosed with an STI or took PrEP [24]. We defined naïve‐PrEP users as individuals who had never heard of or used PrEP, and experienced PrEP users as individuals who used PrEP in the past or were currently using PrEP. The results were presented as coefficients, with a positive coefficient interpreted as a desired attribute level and a negative coefficient interpreted as a less preferred level. The standard deviations (SD) reported from RPL models demonstrate the level of heterogeneity for each attribute level: a statistically significant SD indicates heterogeneity in respondents’ preferences for that attribute level. We calculated each attribute's relative importance using the coefficient range of each attribute divided by the sum of ranges from all attributes [25]. NLOGIT (version 6, Econometric Software Inc., USA) was used for all model estimations.

## 3 RESULTS

Of 62,000 people who clicked on a survey link, 40,038 provided consent. Of these, 23,465 respondents, including 21,943 MSM and 1522 TGW, completed at least one choice set and were included in the analysis. A total of 132,087 choice sets were completed. Ninety per cent (19,644/21,943) of MSM and 93% (1422/1522) of TGW completed all six choice sets in the DCE. Among respondents included in the analyses, the mean age of MSM and TGW was 31.7(±9.6) and 28.1 (±7.0) years, respectively. All TGW were recruited from only MIC, while 66% of MSM were from MIC. Regarding PrEP use, 8% of MSM and 11% of TGW reported taking PrEP in the past but not currently, while 19% of MSM and 35% of TGW reported currently taking PrEP. In the past 6 months, 15% of MSM reported chemsex, 8% reported an STI diagnosis, 9% were involved in sex work, 59% had multiple partners, and 1% shared injecting equipment. Meanwhile, 28% of TGW reported chemsex, 16% reported an STI diagnosis, 34% were involved in paid sex, 54% had multiple partners, and 4% shared injecting equipment in the last 6 months (Table 2).

**Table 2:** Demographic characteristics and risk behaviours of the study population (MSM, *N* = 21,943 and TGW, *N* = 1522)

	MSM		TGW	
	*n*	%	*n*	%
**Mean age**	Mean 31.7	SD 9.6	Mean 28.1	SD 7.0
**Sex at birth**				
Male	21,383	97.5	1357	89.2
Female	151	0.7	40	2.6
Others	409	1.9	125	8.2
**Sexual identity**				
Gay or homosexual	15,123	68.9	631	41.5
Bisexual or pansexual	4703	21.4	196	12.9
Heterosexual or straight	465	2.1	258	17.0
I don't usually use a term	1280	5.8	304	20.0
I use a different term	372	1.7	133	8.7
**Country**				
Asian middle‐income countries	14,529	66.2	1522	100.0
Thailand	1552	7.1	256	16.8
Vietnam	1451	6.6	253	16.6
Indonesia	1428	6.5	80	5.3
The Philippines	2289	10.4	98	6.4
China	1853	8.4	28	1.8
Malaysia	1035	4.7	13	0.9
Myanmar	561	2.6	145	9.5
India	2768	12.6	145	9.5
Cambodia	821	3.7	127	8.3
Laos	312	1.4	34	2.2
Nepal	459	2.1	343	22.5
Asian high‐income countries	7414	33.8	−	−
Taiwan (China)	2564	11.7	−	−
Singapore	770	3.5	−	−
Hong Kong SAR, China	646	2.9	−	−
Japan	1540	7.0	−	−
Australia	1894	8.6	−	−
**Education level**				
No schooling	120	0.6	57	3.8
Up to high school or equivalent	7515	34.3	984	64.7
At least undergraduate degree	13,372	60.9	460	30.2
Other^a^	936	4.3	3	0.2
**Employment**				
Full time	13,931	63.5	617	40.5
Part time	2257	10.3	272	17.9
Unemployed	1894	8.6	318	20.9
Student	2912	13.3	177	11.6
Retired	242	1.1	17	1.1
Other	532	2.4	78	5.1
Missing	175	0.8	43	2.8
**PrEP awareness and use**				
Never heard of PrEP	4214	19.2	300	19.7
Heard of PrEP but never used PrEP	10,961	49.8	457	30.0
Used in the past but not currently	1768	8.1	166	10.9
Currently using PrEP	4098	18.7	531	34.9
Missing	902	4.3	68	4.5
**Chemsex in the last 6 months**				
No sexual partner	838	38.2	59	3.9
No chemsex	15,463	70.5	924	60.7
Had chemsex	3378	15.4	421	27.7
Missing	2264	10.3	118	7.8
**STI diagnosis in the last 6 months**				
No	17,101	77.9	1103	72.5
Yes	1712	7.8	249	16.4
Missing	3130	14.3	170	11.2
**Involvement in commercial sex in the last 6 months**				
No	17,259	78.7	867	57.0
Yes	1989	9.1	518	34.0
Missing	2695	12.3	137	9.0
**Sexual partner in the last 6 months**				
Single	2188	10.0	180	11.8
One partner	3751	17.1	237	15.6
Multiple partners	13,028	59.4	826	54.3
Missing	2976	13.6	149	9.8
**Condomless anal sex in the last 6 months**				
No sex	3481	15.9	210	13.8
Always used condoms	5250	23.9	402	26.4
Not always used condoms	10,094	46.0	742	48.8
Missing	3118	14.2	168	11.0
**Sharing injecting equipment in the last 6 months**				
Not injecting drugs	17,599	80.2	1110	72.9
Injecting drug without sharing equipment	1074	4.9	174	11.4
Injecting drug and sharing equipment	173	0.8	54	3.6
Missing	3097	14.1	175	11.5

Abbreviations: MSM, men who have sex with other men; PrEP, pre‐exposure prophylaxis; SD, standard deviation; STI, sexually transmitted infection; TGW, trans women.

^a^ Diploma/trade/vocational certificate/Singapore education.

**Figure 1:**
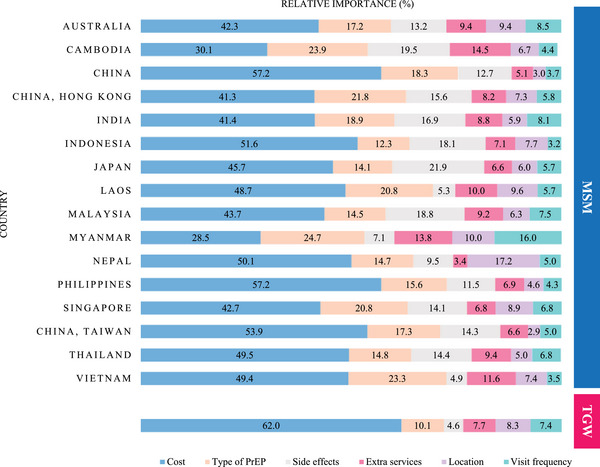
Drivers of choice (relative importance) of PrEP for men who have sex with men (*N* = 21,943) and trans women (*N* = 1522) in Asian countries and Australia.

Among MSM, cost was the most important attribute in all countries, followed by the type of PrEP. There were significant variations across the region regarding the relative importance of other PrEP service attributes, including the side effects of PrEP, service locations and the frequency of clinic visits. However, service locations and clinic visit frequency were generally the least important attributes for PrEP use among MSM (Figure 1 and Figure S1). The coefficients for each country can be found in Tables S1 and S2. The sensitivity analyses conducted for those who completed all choice tasks showed similar results to the main analyses (Table S3).

At least a 50% absolute increase in PrEP uptake was predicted when switching from the least preferred PrEP programmes (including very high cost, PrEP implants, rare kidney problems as side effects and visiting service locations every 2 months) to the optimal PrEP service programme (including free, available in peer‐led community clinics, visiting the clinic every 6 or 12 months and STI testing as an additional service) among MSM in the following countries: Australia, China, Hong Kong SAR China, Japan, the Philippines, Taiwan (China) and Thailand. The details of the predicted uptake of PrEP among MSM, given different PrEP programme configurations in each country, are shown in Table 3.

**Table 3:** The predicted uptake of the worst and best PrEP service package among MSM and TGW in 15 Asian countries and Australia

	Scenario	Uptake (%)	Increase in uptake (%)^a^	Type of PrEP	Service location	Cost	Side effects	Visit frequency	Extra service
**TGW**									
Asia (*N* = 1522)	Worst	50		Implant	Hospital	Very high	Rare chance of kidney problems	Every 2 months	None
	Status quo	60	10						
	Best	87	37	Injectable	Community clinic run by MSM/TG	Free	No side effects	Every 6 or 12 months	STI testing
**MSM**									
Australia (*N* = 1892)	Worst	48		Implant	Hospital	Very high	Rare chance of kidney problems	Every 2 months	None
	Status quo	86	38						
	Best	100	52	Long‐acting oral	Pharmacy	Free	No side effects	Once a year	STI testing
Cambodia (*N* = 821)	Worst	67		Implant	Pharmacy	Very high	Rare chance of kidney problems	Every 2 months	Mental health counselling
	Status quo	88	21						
	Best	97	30	On‐demand	Private community clinic (incl. GP)	Free	No side effects	Every 3 months	STI testing
China (*N* = 1850)	Worst	49		Implant	STI clinic	Very high	Rare chance of kidney problems	Every 2 months	Mental health counselling
	Status quo	87	38						
	Best	100	51	On‐demand	Private community clinic (incl. GP)	Free	No side effects	Once a year	STI testing
Hong Kong SAR, China (*N* = 645)	Worst	37		Implant	STI clinic	Very high	Rare chance of kidney problems	Every 2 months	Mental health counselling
	Status quo	96	59						
	Best	100	63	Long‐acting oral	Telehealth	Free	Mild pain at the injection site	Once a year	STI testing
India (*N* = 2765)	Worst	48		Implant	Telehealth	Very high	Rare chance of kidney problems	Every 2 months	None
	Status quo	80	32						
	Best	95	47	Long‐acting oral	Community clinic run by MSM/TG	Free	No side effects	Once a year	STI testing
Indonesia (*N* = 1427)	Worst	76		Daily oral	Hospital	Very high	Rare chance of kidney problems	Every 2 months	Mental health counselling
	Status quo	91	15						
	Best	100	24	On‐demand	Community clinic run by MSM/TG	Free	No side effects	Every 6 months	STI testing
Japan (*N* = 1540)	Worst	47		Implant	STI clinic	Very high	Rare chance of kidney problems	Every 2 months	Mental health counselling
	Status quo	94	47						
	Best	100	53	Long‐acting oral	Community clinic run by MSM/TG	Free	No side effects	Every 6 months	STI testing
Laos (*N* = 312)	Worst	92		Implant	Pharmacy	Very high	Mild	Once a year	None
	Status quo	92	0						
	Best	100	8	On‐demand	Private community clinic (incl. GP)	Free	Mild pain at the injection site	Every 3 months	STI testing
Malaysia (*N* = 1034)	Worst	78		Implant	Private community clinic (incl. GP)	Very high	Rare chance of kidney problems	Every 2 months	Mental health counselling
	Status quo	93	15						
	Best	100	22	On‐demand	Community clinic run by MSM/TG	Free	No side effects	Once a year	STI testing
Myanmar (*N* = 561)	Worst	97		Implant	Hospital	Very high	Rare chance of kidney problems	Every 2 months	None
	Status quo	99	2						
	Best	100	3	On‐demand	Community clinic run by MSM/TG	Free	No side effects	Every 6 months	STI testing
Nepal (*N* = 459)	Worst	70		Implant	Private community clinic (incl. GP)	Very high	Rare chance of kidney problems	Once a year	STI testing
	Status quo	86	16						
	Best	100	30	Injection	Community clinic run by MSM/TG	Free	No side effects	Every 6 months	None
Philippines (*N* = 2285)	Worst	48		Implant	Telehealth	Very high	Rare chance of kidney problems	Every 6 months	Mental health counselling
	Status quo	89	41						
	Best	100	52	On‐demand	Community clinic run by MSM/TG	Free	No side effects	Once a year	STI testing
Singapore (*N* = 769)	Worst	76		Implant	STI clinic	Very high	Interactions with other medications	Every 2 months	Mental health counselling
	Status quo	96	20						
	Best	100	24	Long‐acting oral	Community clinic run by MSM/TG	Free	No side effects	Once a year	STI testing
Taiwan (China) (*N* = 2506)	Worst	20		Implant	STI clinic	Very high	Rare chance of kidney problems	Every 2 months	Mental health counselling
	Status quo	86	66						
	Best	100	80	On‐demand	Community clinic run by MSM/TG	Free	No side effects	Once a year	STI testing
Thailand (*N* = 1551)	Worst	44		Implant	Hospital	Very high	Interactions with other medications	Every 2 months	Mental health counselling
	Status quo	87	43						
	Best	99	55	Long‐acting oral	Community clinic run by MSM/TG	Free	No side effects	Every 6 months	STI testing
Vietnam (*N* = 1451)	Worst	80		Implant	Telehealth	Very high	Mild	Every 2 months	Mental health counselling
	Status quo	94	14						
	Best	99	19	Daily oral	Community clinic run by MSM/TG	Free	No side effects	Every 3 months	None

Abbreviations: MSM, men who have sex with other men; PrEP, pre‐exposure prophylaxis; STI, sexually transmitted infection; TG, trans women.

^a^ %increase in uptake is calculated from the difference in uptake between the best and the worst PrEP programme.

The best PrEP programmes included attribute levels most preferred by MSM or TGW in each country.

The worst PrEP programmes included attribute levels least preferred by MSM or TGW in each country.

Status quo programmes included daily oral PrEP, access via hospital, high‐cost PrEP, mild side effects, clinic visit every 3 months and no additional service.

**Figure 2:**
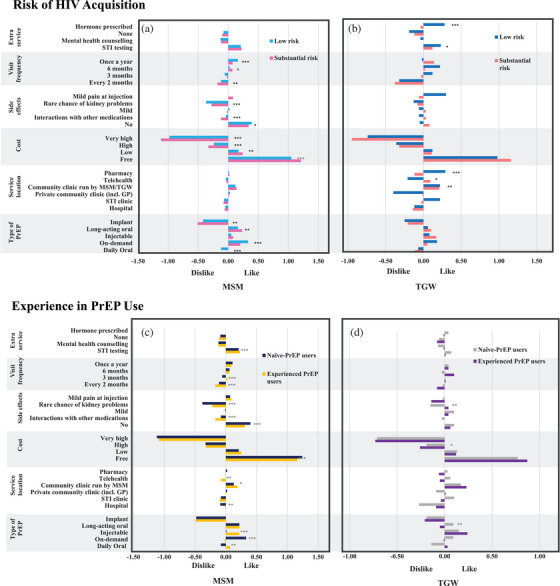
Heterogeneity preference for PrEP among MSM and TGW. *significant at *p*<0.10, **significant at *p*<0.05, ***significant at *p*<0.01. Abbreviations: GP, general practice; MSM, men who have sex with men; STI, sexually transmitted infection; TGW, transgender women.

When examining preference for PrEP service programmes among MSM with different levels of risk of HIV, MSM at substantial risk (15,841/21,943) had a greater preference for free long‐acting oral PrEP (*p* = 0.0217) and visiting the service location every 6 months (*p* = 0.0469) compared to the low‐risk group (6102/21,943) who had a greater preference for PrEP without side effects (*p* = 0.0475) and preferred to visit a service location once a year (*p* = 0.0010). The two groups showed no significant difference in preferences for service locations and extra services (Figure 2A,C and Table S4). When comparing preferences for PrEP service programmes between MSM who were experienced PrEP users (5866/21,041) and naïve PrEP users (15,175/21,041), naïve PrEP users were more likely to use PrEP if the programme offered free on‐demand PrEP (*p*<0.0001) with no side effects (*p*<0.0001), and the option for telehealth access (*p* = 0.0014) (Figure 2 and Table S5).

When examining drivers of PrEP use among TGW, the most important attribute of PrEP use was cost (62% relative importance), followed by the type of PrEP (10% relative importance), location (8% relative importance), extra services (8% relative importance), visit frequency (7% relative importance) and side effects (5% relative importance) (Figure 1). We predicted a 37% absolute increase in PrEP uptake when switching from the least preferred PrEP service (including very high cost, PrEP implants, a rare chance of kidney problems as side effects, a hospital as a service location, visits every 2 months and no additional services) to the optimal PrEP service configuration (including no cost, injectable PrEP, no side effects, a peer‐led clinic as a service location, 6 or 12 monthly visit and additional STI testing services) (Table 3 and Figure S2). The sensitivity analyses conducted for those who completed all choice tasks showed similar results to the main analyses (Table S3).

When exploring preferences for PrEP service programmes among TGW with different risk profiles for HIV, TGW at substantial risk had a greater preference to access PrEP through telehealth (*p* = 0.0569) with additional STI testing (*p* = 0.0802). In contrast, low‐risk TGW had a greater preference to access PrEP through a pharmacy (*p* = 0.0080) with gender‐affirming prescription hormones (*p* = 0.0048) as an extra service. They had less preference to access PrEP through a private community clinic (*p* = 0.0124) compared to the group at higher risk (Figure 2B,D and Table S6). When comparing preferences for PrEP service programmes between experienced PrEP users (697/1454) and naïve PrEP users (757/1454) among TGW, naïve‐PrEP users had less preference to use PrEP with a rare chance of kidney problems (*p* = 0.0404) and preferred long‐acting oral PrEP (*p* = 0.0190) (Figure 2 and Table S7).

## 4 DISCUSSION

This study of PrEP preferences among MSM and TGW in 15 Asian countries and territories and Australia highlighted that cost was the key driver of PrEP, despite diverse preferences across countries. Tailoring PrEP service configurations to country contexts was predicted to substantially enhance PrEP uptake. The observed differences in service attribute importance signified the influence of local contexts on PrEP use. This study offered valuable preference data for advocacy and evidence‐based policies to improve PrEP uptake in key populations in each Asian country or territory and Australia, ultimately contributing to ending HIV transmission in the region.

Accessing PrEP at no cost emerged as the most important driver of PrEP use among MSM and TGW, irrespective of country, HIV risk or prior PrEP experience. Cost remains a major barrier when PrEP is not covered by national health systems, social security or insurance [26, 27]. Additional expenses, such as consultations, HIV/STI testing, transportation and taking time off work, must be factored in as they impede access to PrEP [28]. Offering free access could proactively attract individuals who may benefit most. Given the enormous lifetime costs of managing HIV [29], a country's investment in free or affordable access to PrEP could be cost‐effective or even cost‐saving in the HIV response [30].

Our findings revealed that, in most countries, MSM and TGW preferred accessing PrEP through peer‐led community clinics. This aligns with the 2021 United Nations General Assembly's Political Declaration on HIV and AIDS, which encourages expanding community‐delivered HIV services [31]. Community‐led PrEP services, an option under differentiated service delivery [12], have successfully scaled up PrEP in several Asian countries, including the Philippines, Thailand and Vietnam [32, 33, 34, 35]. To ensure the sustainability of community‐led services and accelerate PrEP access, it is essential to endorse, accredit, fund and integrate them within the national HIV response. Within community‐led services, lay and community health providers must be trained and supported, linked to healthcare facilities, given clinical oversight during transitions and receive appropriate remuneration to deliver high‐quality services to reach communities in need [36].

Another driver of PrEP use within governments’ purview is subsidizing additional services. Approximately one in four individuals who would benefit from PrEP had an STI diagnosis [37]. Integrating STI testing into PrEP services allows individuals to access HIV/STIs prevention in one setting [37], improving operational efficiencies, care quality and adherence to PrEP, and promoting person‐centred care [38]. However, financial support from government bodies is required for training, supporting the workers and supplying the tests, ensuring the long‐term sustainability of the programmes [37]. Further, given that most MSM and TGW in this study preferred an annual clinic visit, STI and HIV self‐testing should be explored as an alternative to conventional testing.

The significant predicted increase in PrEP uptake from country‐specific optimized programmes underscores the importance of comprehensive, person‐centred PrEP approaches. Our findings align with Andersen's Behavioral Model, where predisposing characteristics, enabling resources and perceived need influence service use [39]. Preferences for free PrEP services and fewer visits reflect reduced access barriers, while varied preferences for PrEP type and service delivery suggest individual and contextual influences. Designing PrEP services around local preferences enables decision‐makers to allocate resources efficiently and focus on the areas where policy changes are essential. For instance, providing a free 1‐year supply of long‐acting oral PrEP with STI testing through pharmacies was predicted to substantially improve PrEP uptake among MSM in Australia, while peer‐led community clinics were preferred by most Asian countries. Similar studies reported that injectable or implant PrEP was preferred by MSM in Brazil [19], while no‐cost injectable PrEP was preferred in the United States [40]. In contrast, another DCE of 675 MSM showed that nearly 50% had no specific preference for PrEP service attributes [41]. Tailoring PrEP service programmes can be costly, but they are vital for ending HIV transmission. Additionally, multipronged strategies to reduce HIV‐related stigma, discrimination and cultural beliefs should be integrated into the programmes to create a more inclusive and accepting society for PrEP users. Further qualitative research is needed to explore these issues in greater depth and inform the development of interventions.

Our study has several strengths. First, this is one of the largest DCE surveys globally, with over 23,000 participants, providing precise estimated parameters and region‐level preferences. Second, our country‐level data recognized that preferences are shaped by local socio‐cultural and economic contexts. Third, our quantitative data about the demand for service delivery models is valuable for decision‐makers to optimize PrEP uptake among people who would benefit most. Our study has limitations. First, the use of non‐probabilistic sampling limits generalizability to all MSM and TGW. Second, as an online survey, dropouts were common due to survey length, perceived complexity or personal time constraints. Further, MSM and TGW face specific challenges, such as stigma and privacy concerns, which may further contribute to attrition. We minimized dropouts through incentives, simple survey design and clear instructions. Third, there could be unavoidable sampling bias as we lacked data on non‐respondents who may have different preferences. Fourth, small TGW sample sizes in several countries (e.g. in Laos and China) required combining TGW data into one model. During our initial feasibility assessment of which countries to include TGW populations, we did not identify sufficiently well‐established community organizations in HIC that could effectively reach the networks of TGW. However, the inclusion of TGW was important to capture preliminary data on their PrEP service preferences, which are rarely captured in existing research. Fifth, hypothetical bias is inherent in stated preference surveys [14]. This could lead to overstated willingness to use PrEP compared to real‐life behaviour. Further studies are needed to evaluate the acceptability, feasibility and cost‐effectiveness of each country's various service delivery models. Sixth, we were unable to conduct sensitivity analyses related to completion speed or straightlining, as our survey platform did not record the duration of choice task completion. Seventh, external validity is a common concern, although there is strong evidence for the external validity of DCEs [14, 42, 43]. Last, we did not conduct qualitative interviews to identify attributes and levels included. Instead, we relied on our systematic review [16], advice from community and experts, and a ranking exercise to select the attributes. Although this approach allowed us to compare the relative importance of the same attributes across countries, there may be unique attributes for specific countries that we did not explicitly measure (e.g. provider attitudes, clinic opening hours or privacy).

## 5 CONCLUSIONS

Preference‐sensitive PrEP services can optimize PrEP use and accelerate its uptake among those who may most benefit from PrEP. Choice data can inform evidence‐based policy and effective resource allocation. Investing in community‐based, locally tailored and no‐cost PrEP service could increase equitable access and uptake of PrEP and achieve the ambitious UNAIDS 95‐95‐95 target to end AIDS as a threat to public health by 2030.

## COMPETING INTERESTS

BRB has received unrestricted research grants, honoraria and travel from ViiV Healthcare and Gilead Sciences. Other authors reported no conflict of interest.

## AUTHORS’ CONTRIBUTIONS

JJO, BRB and H‐MAS conceived the idea for the manuscript. CC managed the project. JJO designed the DCE survey. BRB, H‐MAS, KEG, NP, DF, CC, HB, MC, LZ and WTa contributed to the design and delivery of the survey. CC and DF collected the data. CC did the initial data cleaning. WTi conducted the analysis, wrote the first draft of the manuscript, and revised and finalized the manuscript. All authors contributed to the manuscript and approved the final version for submission.

## FUNDING

This study was supported by funding from the World Health Organization, the Kirby Institute and the Outstanding Young Scholars Support Program funds from ViiV Healthcare, the NSW Ministry of Health, the MAC AIDS Fund, and the Australian Government Department of Health supported the Australian arm of the study.

## Supporting information




**Appendix 1**. An example of a discrete choice experiment question
**Table S1**. Preferences of men who have sex with men in middle‐income Asian countries
**Table S2**. Preferences of men who have sex with men in high‐income Asian countries and Australia
**Table S3**. Preferences of men who have sex with men and transwomen who completed all questions.
**Table S4**. PrEP preference between MSM at substantial‐ vs low risk of HIV infection
**Table S5**. PrEP preference between MSM who were experienced‐ vs naive‐PrEP users
**Table S6**. Heterogeneity preference between TGW at substantial‐ vs low‐risk of HIV infection
**Table S7**. Heterogeneity preference between TGW who were experienced‐ vs naive‐PrEP users
**Figure S1**. Relative importance of attributes for PrEP among men who have sex with men in 15 Asian countries and Australia (N = 21,943)
**Figure S2**. Preference for PrEP among transgender women in 11 Asian countries (N = 1,522).

## Data Availability

The data that support the findings of this study are available from the corresponding author upon request.
