# Supplementary material for: Preferences for HIV pre‐exposure prophylaxis among men who have sex with men and trans women in 15 countries and territories in Asia and Australia: a discrete choice experiment
Source: J Int AIDS Soc. 2025 Aug 20;28(8):e70025. doi: 10.1002/jia2.70025 (PMC12367860; doi:10.1002/jia2.70025)
Supplement: Supplementary file 1 — Appendix 1. An example of a discrete choice experiment question Table S1. Preferences of men who have sex with men in middle‐income Asian countries Table S2. Preferences of men who have sex with men in high‐income Asian countries and Australia Table S3. Preferences of men who have sex with men and transwomen who completed all questions. Table S4. PrEP preference between MSM at substantial‐ vs low risk of HIV infection Table S5. PrEP preference between MSM who were experienced‐ vs naive‐PrEP users Table S6. Heterogeneity preference between TGW at substantial‐ vs low‐risk of HIV infection Table S7. Heterogeneity preference between TGW who were experienced‐ vs naive‐PrEP users Figure S1. Relative importance of attributes for PrEP among men who have sex with men in 15 Asian countries and Australia (N = 21,943) Figure S2. Preference for PrEP among transgender women in 11 Asian countries (N = 1,522). [file JIA2-28-e70025-s001.docx]

**Table of Contents**

|  | **Page** |
| --- | --- |
| Appendix 1. An example of a discrete choice experiment question | 2 |
| Table S1. Preferences of men who have sex with men in middle-income Asian countries | 4 |
| Table S2. Preferences of men who have sex with men in high-income Asian countries and Australia | 6 |
| Table S3. Preferences of men who have sex with men and transwomen who completed all questions. | 7 |
| Table S4. PrEP preference between MSM at substantial- vs low risk of HIV infection | 8 |
| Table S5. PrEP preference between MSM who were experienced- vs naive-PrEP users | 9 |
| Table S6. Heterogeneity preference between TGW at substantial- vs low-risk of HIV infection | 10 |
| Table S7. Heterogeneity preference between TGW who were experienced- vs naive-PrEP users | 11 |
| Figure S1. Relative importance of attributes for PrEP among men who have sex with men in 15 Asian countries and Australia (N=21,943) | 12 |
| Figure S2. Preference for PrEP among transgender women in 11 Asian countries (N=1,522) | 13 |

**Appendix 1. An example of a discrete choice experiment question**

*We will show you a series of six choices with two different options of PrEP. Each PrEP choice is described using the following features.*

- ***Type of PrEP*** *(Daily oral, on-demand oral, injectable, long-acting oral, implant)*
- ***Location*** *to access PrEP (Hospital, STI clinic, Private community clinic (including GP), Community clinic run by peers, Telehealth, Pharmacy)*
- ***Out of pocket cost*** *per month (includes drug cost, tests and consultations)*
- ***Side effects*** *(*No side effects, could interact with other medications (incl. hormones), mild side effects less than 1 month – nausea, headache, rare chance of kidney problems, mild pain at injection site (only for injectable)
- ***PrEP visit frequency*** *(every 2 months, every 3 months, every 6 months, once a year)*
- ***Extra services available*** *(*Comprehensive STI testing (syphilis/chlamydia/gonorrhoea), Gender-affirming hormones prescribed (TG), Mental health counselling, No other extra services (Only PrEP))

*In the example below, choosing option 1 means you prefer an oral pill that you must take daily, cost you $30 per month and has mild side effects. You will have a 6 monthly check up and access your pills from a pharmacy. Choosing option 2 means you prefer to take PrEP on-demand (i.e. only when you are having sex), cost you $100 per month, and has mild side effects. You will have a 3 monthly check up which includes comprehensive STI testing, and access your PrEP in a community clinic run by MSM/TG. If you do not like option 1 or option 2, you can choose “None of these options”.*


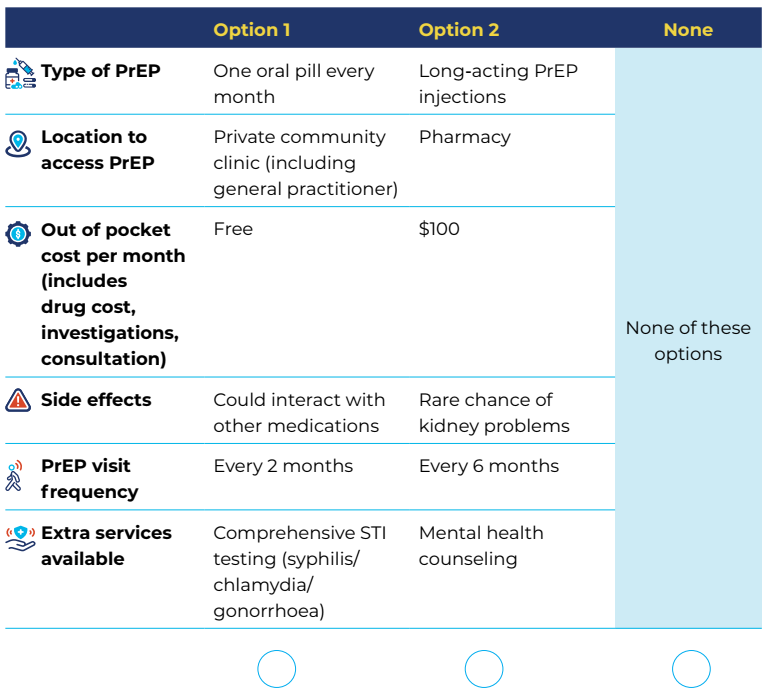


Reference: PrEP APPEAL report (https://www.kirby.unsw.edu.au/research/projects/prep-appeal)

| **Table S1. Preferences of men who have sex with men in middle-income Asian countries** | | | | | | | | | | | | | |
| --- | --- | --- | --- | --- | --- | --- | --- | --- | --- | --- | --- | --- | --- |
|  |  | **Cambodia (N=821)** | | **China (N=1,850)** | | **India (N=2,765)** | | **Indonesia (N=1,427)** | | **Laos (N=312)** | | **Malaysia (N=1,034)** | |
| **Attribute** | **Level** | **Coefficient** | **SD** | **Coefficient** | **SD** | **Coefficient** | **SD** | **Coefficient** | **SD** | **Coefficient** | **SD** | **Coefficient** | **SD** |
| Type of PrEP | Daily Oral | 0.08 | 0.88*** | -0.21*** | 1.67*** | 0.03 | 0.96*** | -0.16** | 1.23*** | -0.19 | 1.17* | -0.12 | 1.54*** |
|  | On-demand | 0.27*** | 0.41*** | 0.57*** | 0.65*** | 0.11*** | 0.48*** | 0.46*** | 0.45*** | 0.39*** | 0.10 | 0.28*** | 0.77*** |
|  | Injectable | 0.14* | 0.45*** | 0.01 | 1.11*** | 0.00 | 0.41*** | 0.18** | 0.57*** | 0.32** | 0.67*** | 0.09 | 0.93*** |
|  | Long-acting oral | -0.05 | 0.46*** | 0.29*** | 0.33* | 0.22*** | 0.33*** | 0.12** | 0.46*** | -0.04 | 0.41 | 0.21*** | 0.48*** |
|  | Implant | -0.44*** | 0.44*** | -0.66*** | 1.02*** | -0.36*** | 0.65*** | -0.6*** | 0.88*** | -0.48*** | 0.86*** | -0.46*** | 0.83*** |
| Location | Hospital | 0.09 | 0.84** | 0.03 | 0.98*** | -0.04 | 0.62** | -0.15** | 0.85** | 0.13 | 1.17 | -0.10 | 0.93 |
|  | STI clinic | -0.06 | 0.50*** | -0.13** | 0.53*** | -0.06 | 0.35*** | 0.06 | 0.42*** | -0.09 | 0.81*** | 0.04 | 0.18 |
|  | Private community clinic (incl. GP) | 0.10 | 0.11 | 0.07 | 0.11 | 0.03 | 0.03 | 0.10 | 0.03 | 0.19 | 0.07 | -0.13 | 0.05 |
|  | Community clinic run by MSM/TG | 0.00 | 0.66*** | 0.02 | 0.60*** | 0.09** | 0.50*** | 0.24*** | 0.61*** | -0.11 | 0.82*** | 0.19** | 0.45** |
|  | Telehealth | -0.02 | 0.04 | 0.01 | 0.47** | -0.09** | 0.06 | -0.05 | 0.17 | 0.09 | 0.05 | -0.09 | 0.38** |
|  | Pharmacy | -0.11 | 0.03 | 0.00 | 0.31* | 0.07* | 0.11 | -0.2*** | 0.37 | -0.21 | 0.15 | 0.09 | 0.69*** |
| Cost | Free | 0.53*** | 0.66*** | 1.97*** | 1.85*** | 0.65*** | 0.90*** | 1.36*** | 1.48*** | 1.15*** | 1.09** | 1.17*** | 1.31*** |
|  | Low | 0.01 | 0.35*** | 0.46*** | 0.58*** | 0.07** | 0.23* | 0.22*** | 0.64*** | 0.14 | 0.04 | 0.19*** | 0.23 |
|  | High | -0.15*** | 0.09 | -0.56*** | 0.72*** | -0.10*** | 0.22 | -0.34*** | 0.67*** | -0.40*** | 0.49** | -0.30*** | 0.55*** |
|  | Very high | -0.39*** | 0.55*** | -1.87*** | 1.60*** | -0.62*** | 0.84*** | -1.24*** | 1.16*** | -0.89*** | 0.97*** | -1.06*** | 1.17*** |
| Side effects | No | 0.41*** | 0.67** | 0.49*** | 0.78* | 0.27*** | 0.57** | 0.37 | 0.90*** | -0.05 | 0.97* | 0.58*** | 0.83** |
|  | Interactions with other medications | -0.10 | 0.41*** | -0.10* | 0.43*** | -0.11*** | 0.29*** | 0.12 | 0.25* | 0.00 | 0.37 | -0.11 | 0.14 |
|  | Mild | 0.00 | 0.09 | -0.23*** | 0.50*** | 0.16*** | 0.19 | 0.10* | 0.19* | -0.08 | 0.51** | -0.01 | 0.27* |
|  | Rare chance of kidney problems | -0.17** | 0.35** | -0.36*** | 0.29 | -0.25*** | 0.43*** | -0.54*** | 0.66*** | -0.01 | 0.57*** | -0.38*** | 0.65*** |
|  | Mild pain at injection | -0.14 | 0.39** | 0.20* | 0.30 | -0.07 | 0.14 | -0.05 | 0.52*** | 0.14 | 0.46 | -0.08 | 0.41 |
| Visit frequency | Every 2 months | -0.06 | 0.35 | -0.15** | 0.63*** | -0.09** | 0.18 | -0.09 | 0.49** | -0.06 | 0.44 | -0.17** | 0.46 |
|  | 3 months | 0.07 | 0.12 | -0.04 | 0.21 | -0.06** | 0.02 | -0.03 | 0.14 | 0.11 | 0.09 | -0.08 | 0.09 |
|  | 6 months | -0.03 | 0.31** | 0.09* | 0.40*** | -0.01 | 0.17* | 0.07 | 0.47*** | 0.08 | 0.43* | 0.04 | 0.39*** |
|  | Once a year | 0.02 | 0.12 | 0.10** | 0.44*** | 0.16*** | 0.04 | 0.05 | 0.02 | -0.13 | 0.01 | 0.21*** | 0.23 |
| Extra services | STI testing | 0.28*** | 0.47*** | 0.20*** | 0.56*** | 0.18*** | 0.46*** | 0.23*** | 0.48*** | 0.21** | 0.74*** | 0.29*** | 0.52*** |
|  | Mental health counselling | -0.15*** | 0.40*** | -0.14*** | 0.45*** | -0.09*** | 0.39*** | -0.13*** | 0.11 | 0.00 | 0.55*** | -0.18*** | 0.29* |
|  | None | -0.13*** | 0.24** | -0.06 | 0.34*** | -0.09*** | 0.25*** | -0.10** | 0.47*** | -0.21*** | 0.49*** | -0.11** | 0.43*** |
| Neither |  | -1.34*** | 5.52*** | -2.37*** | 5.14*** | -0.71*** | 5.05*** | -2.53*** | 4.82*** | -3.69*** | 5.07*** | -2.96*** | 4.41*** |
| *significant at P<0.10, **significant at P<0.05, ***significant at P<0.01  GP=general practice, MSM=men who have sex with other men, SD=Standard Deviation, STI=sexually transmitted infection | | | | | | | | | | | |  |  |

| **Table S1 (cont). Preferences of men who have sex with men in middle-income Asian countries** | | | | | | | | | | | |
| --- | --- | --- | --- | --- | --- | --- | --- | --- | --- | --- | --- |
|  |  | **Myanmar (N=561)** | | **Nepal (N=459)** | | **Philippines (N=2,285)** | | **Thailand (N=1,551)** | | **Vietnam (N=1,451)** | |
| **Attribute** | **Level** | **Coefficient** | **SD** | **Coefficient** | **SD** | **Coefficient** | **SD** | **Coefficient** | **SD** | **Coefficient** | **SD** |
| Type of PrEP | Daily Oral | -0.10 | 1.81*** | 0.01 | 1.49*** | -0.10 | 1.81*** | 0.03 | 1.74*** | 0.31*** | 1.87*** |
|  | On-demand | 0.40*** | 0.88*** | 0.13 | 0.85*** | 0.40*** | 0.88*** | 0.15** | 0.77*** | 0.12* | 1.07*** |
|  | Injectable | 0.13 | 1.07*** | 0.21 | 0.67*** | 0.13 | 1.07*** | 0.10 | 0.98*** | 0.23*** | 0.84*** |
|  | Long-acting oral | 0.08 | 0.97*** | 0.09 | 0.86*** | 0.08 | 0.97*** | 0.26*** | 0.64*** | 0.04 | 0.74*** |
|  | Implant | -0.51*** | 0.65*** | -0.44*** | 0.56** | -0.51*** | 0.65*** | -0.54*** | 1.04*** | -0.70*** | 1.04*** |
| Location | Hospital | -0.17 | 0.96* | 0.02 | 1.36** | -0.17 | 0.96* | -0.15* | 1.07*** | -0.03 | 1.03*** |
|  | STI clinic | 0.10 | 0.67*** | 0.10 | 0.73*** | 0.10 | 0.67*** | -0.09 | 0.67*** | -0.07 | 0.83*** |
|  | Private community clinic (incl. GP) | -0.14 | 0.04 | -0.43*** | 0.12 | -0.14 | 0.04 | 0.11 | 0.20 | 0.05 | 0.07 |
|  | Community clinic run by MSM | 0.20** | 0.68*** | 0.33** | 1.14*** | 0.20** | 0.68*** | 0.12* | 0.51*** | 0.18*** | 0.51*** |
|  | Telehealth | 0.02 | 0.06 | -0.03 | 0.01 | 0.02 | 0.06 | 0.08 | 0.62*** | -0.14*** | 0.28* |
|  | Pharmacy | -0.01 | 0.02 | 0.01 | 0.00 | -0.01 | 0.02 | -0.07 | 0.11 | 0.01 | 0.15 |
| Cost | Free | 0.61*** | 0.66** | 1.39*** | 1.43*** | 0.61*** | 0.66** | 1.38*** | 1.45*** | 1.16*** | 1.34*** |
|  | Low | -0.02 | 0.34 | -0.14 | 0.86*** | -0.02 | 0.34 | 0.20*** | 0.24 | 0.20*** | 0.09 |
|  | High | -0.15* | 0.52*** | -0.43*** | 0.70*** | -0.15* | 0.52*** | -0.28*** | 0.53*** | -0.38*** | 0.66*** |
|  | Very High | -0.44*** | 0.23 | -0.82*** | 0.90*** | -0.44*** | 0.23 | -1.30*** | 1.33*** | -0.98*** | 1.16*** |
| Side effects | No | 0.15 | 1.11** | 0.23 | 1.31*** | 0.15 | 1.11** | 0.34*** | 0.87** | 0.14*** | 0.77** |
|  | Interactions with other medications | 0.01 | 0.79*** | -0.09 | 0.58*** | 0.01 | 0.79*** | -0.44*** | 0.55*** | -0.07 | 0.62*** |
|  | Mild | -0.06 | 0.23 | 0.07 | 0.64*** | -0.06 | 0.23 | 0.17*** | 0.04 | -0.07 | 0.34* |
|  | Rare chance of kidney problems | -0.11 | 0.62*** | -0.19 | 0.58*** | -0.11 | 0.62*** | -0.12** | 0.48*** | 0.01 | 0.30* |
|  | Mild pain at injection | 0.01 | 0.41 | -0.02 | 0.80** | 0.01 | 0.41 | 0.05 | 0.47** | -0.01 | 0.00 |
| Visit frequency | Every 2 months | -0.30*** | 0.39 | -0.04 | 0.85* | -0.30*** | 0.39 | -0.14** | 0.55** | -0.08 | 0.41 |
|  | 3 months | 0.00 | 0.03 | -0.04 | 0.06 | 0.00 | 0.03 | -0.04 | 0.20 | 0.07 | 0.02 |
|  | 6 months | 0.29*** | 0.39* | 0.15 | 0.84*** | 0.29*** | 0.39* | 0.23*** | 0.46*** | 0.00 | 0.38*** |
|  | Once a year | 0.01 | 0.05 | -0.07 | 0.13 | 0.01 | 0.05 | -0.05 | 0.22 | 0.01 | 0.15 |
| Extra services | STI testing | 0.29*** | 0.71*** | -0.09 | 1.00*** | 0.29*** | 0.71*** | 0.32*** | 0.57*** | 0.05 | 0.64*** |
|  | Mental health counselling | -0.07 | 0.53*** | 0.03 | 0.67*** | -0.07 | 0.53*** | -0.19*** | 0.46*** | -0.11*** | 0.56*** |
|  | None | -0.22*** | 0.47*** | 0.06 | 0.74*** | -0.22*** | 0.47*** | -0.13*** | 0.34*** | 0.06 | 0.30*** |
| Neither |  | -4.58*** | 5.21*** | -2.17*** | 5.04*** | -4.58*** | 5.21*** | -1.80*** | 5.15*** | -0.78*** | 4.53 |
| *significant at P<0.10, **significant at P<0.05, ***significant at P<0.01 | | | | | | | | | | | |

GP=general practice, MSM=men who have sex with other men, SD=Standard Deviation, STI=sexually transmitted infection

| **Table S2. Preferences of men who have sex with men in high-income Asian countries and Australia** | | | | | | | | | | | |
| --- | --- | --- | --- | --- | --- | --- | --- | --- | --- | --- | --- |
|  |  | **Hongkong (N=645)** | | **Japan (N=1,540)** | | **Singapore (N=769)** | | **Taiwan (N=2,506)** | | **Australia (N=1,892)** | |
| **Attribute** | **Level** | **Coefficient** | **SD** | **Coefficient** | **SD** | **Coefficient** | **SD** | **Coefficient** | **SD** | **Coefficient** | **SD** |
| Type of PrEP | Daily Oral | 0.21 | 1.33*** | -0.05 | 1.73*** | -0.02 | 1.83*** | -0.05 | 1.55*** | -0.01 | 2.05*** |
|  | On-demand | 0.28** | 1.03*** | 0.36*** | 0.83*** | 0.25*** | 0.84*** | 0.71*** | 0.73*** | -0.29*** | 1.06*** |
|  | Injectable | -0.25* | 0.41* | -0.12* | 0.87*** | 0.06 | 1.19*** | -0.19*** | 0.90*** | 0.26*** | 1.04*** |
|  | Long-acting oral | 0.63*** | 0.33 | 0.40*** | 0.76*** | 0.38*** | 0.39** | 0.26*** | 0.59*** | 0.56*** | 0.49*** |
|  | Implant | -0.87*** | 0.65*** | -0.59*** | 0.99*** | -0.67*** | 1.04*** | -0.73*** | 0.84*** | -0.52*** | 1.32*** |
| Location | Hospital | -0.24 | 0.62 | -0.09 | 1.09*** | -0.09 | 0.62 | 0.01 | 0.79* | -0.33*** | 0.99 |
|  | STI clinic | -0.26** | 0.35 | -0.21*** | 0.54*** | -0.15* | 0.00 | -0.14*** | 0.02 | -0.11* | 0.55*** |
|  | Private community clinic (incl. GP) | 0.18 | 0.03 | -0.13* | 0.26 | 0.02 | 0.07 | 0.04 | 0.06 | 0.08 | 0.19 |
|  | Community clinic run by MSM | 0.10 | 0.50** | 0.21*** | 0.52*** | 0.30*** | 0.57*** | 0.10*** | 0.52*** | 0.20*** | 0.00 |
|  | Telehealth | 0.24** | 0.06 | 0.06 | 0.73*** | -0.15* | 0.13 | -0.07 | 0.58*** | -0.10 | 0.70*** |
|  | Pharmacy | -0.02 | 0.11 | 0.16** | 0.17 | 0.07 | 0.19 | 0.06 | 0.14 | 0.26*** | 0.38** |
| Cost | Free | 1.32*** | 0.90** | 1.52*** | 1.23*** | 1.05*** | 1.02*** | 2.41*** | 1.87*** | 1.28*** | 1.11*** |
|  | Low | 0.29*** | 0.23 | 0.41*** | 0.10 | 0.22*** | 0.28 | 0.49*** | 0.42*** | 0.45*** | 0.01 |
|  | High | -0.09 | 0.22 | -0.24*** | 0.69*** | -0.17*** | 0.37** | -0.82*** | 1.07*** | -0.36*** | 0.46*** |
|  | Very high | -1.52*** | 0.84*** | -1.69*** | 1.01*** | -1.10*** | 0.91*** | -2.08*** | 1.48*** | -1.37*** | 1.01*** |
| Side effects | No | 0.35*** | 0.57 | 0.78*** | 1.05*** | 0.42*** | 0.76* | 0.62*** | 0.85*** | 0.48*** | 0.75** |
|  | Interactions with other medications | -0.05 | 0.29 | -0.04 | 0.21 | -0.29*** | 0.31* | -0.12** | 0.43*** | -0.24*** | 0.31** |
|  | Mild | -0.11 | 0.19 | -0.17*** | 0.64*** | 0.11 | 0.02 | -0.14*** | 0.25* | -0.07 | 0.13 |
|  | Rare chance of kidney problems | -0.63*** | 0.43** | -0.76*** | 0.77*** | -0.28*** | 0.69*** | -0.57*** | 0.68*** | -0.35*** | 0.67*** |
|  | Mild pain at injection | 0.44** | 0.14 | 0.19* | 0.23 | 0.04 | 0.04 | 0.21** | 0.08 | 0.18** | 0.06 |
| Visit frequency | Every 2 months | -0.19* | 0.18 | -0.26*** | 0.28 | -0.20*** | 0.22 | -0.23*** | 0.67*** | -0.32*** | 0.43** |
|  | 3 months | -0.12 | 0.02 | 0.01 | 0.10 | 0.01 | 0.07 | -0.06 | 0.20 | -0.01 | 0.02 |
|  | 6 months | 0.10 | 0.17 | 0.14*** | 0.26* | 0.05 | 0.20 | 0.10** | 0.48*** | 0.12*** | 0.28** |
|  | Once a year | 0.21** | 0.04 | 0.11** | 0.06 | 0.14** | 0.04 | 0.19*** | 0.43*** | 0.21*** | 0.33** |
| Extra services | STI testing | 0.30*** | 0.46* | 0.26*** | 0.55*** | 0.21*** | 0.48*** | 0.32*** | 0.71*** | 0.38*** | 0.56*** |
|  | Mental health counselling | -0.26*** | 0.39* | -0.20*** | 0.42*** | -0.13*** | 0.34*** | -0.23*** | 0.64*** | -0.17*** | 0.37*** |
|  | None | -0.04 | 0.25 | -0.06 | 0.35*** | -0.08 | 0.34*** | -0.09*** | 0.30*** | -0.21*** | 0.42*** |
| Neither |  | -2.52*** | 5.05*** | -2.91*** | 4.83*** | -3.00*** | 4.27*** | -1.88*** | 4.46*** | -2.30*** | 0.35** |
| *significant at P<0.10, **significant at P<0.05, ***significant at P<0.01  GP=general practice, MSM=men who have sex with other men, SD=Standard Deviation, STI=sexually transmitted infection | | | | | | | | | | | |

**Table S3. Preferences of men who have sex with men and transwomen who completed all questions.**

| **Attribute** | **Level** | **MSM**  **(n=19,644)** | | **TGW**  **(n=1,422)** | |
| --- | --- | --- | --- | --- | --- |
|  |  | **Coefficient** | **SD** | **Coefficient** | **SD** |
| Type of PrEP | Daily Oral* | -0.03* | 1.15*** | -0.11* | 3.93*** |
|  | On-demand | 0.22*** | 0.06** | 0.04 | 1.32 |
|  | Injectable** | 0.08*** | 0.71*** | 0.17** | 8.11*** |
|  | Long-acting oral** | 0.22*** | 0.44*** | 0.10** | 4.08*** |
|  | Implant*** | -0.49*** | 0.78*** | -0.20*** | 3.42*** |
| Location | Hospital** | -0.06*** | 0.63*** | -0.13** | 1.49 |
|  | STI clinic | -0.08*** | 0.41*** | 0.01 | 3.50*** |
|  | Private community clinic (incl. GP) | 0.02 | 0.03 | -0.07 | 0.61 |
|  | Community clinic run by TGW*** | 0.13*** | 0.46*** | 0.20*** | 2.92*** |
|  | Telehealth | -0.03* | 0.13 | 0.04 | 1.50 |
|  | Pharmacy | 0.01 | 0.02 | -0.05 | 0.14 |
| Cost | Free*** | 1.20*** | 1.20*** | 1.15*** | 7.83*** |
|  | Low cost** | 0.22*** | 0.19*** | 0.09** | 3.40*** |
|  | High cost*** | -0.31*** | 0.50*** | -0.32*** | 6.88*** |
|  | Very high cost*** | -1.11*** | 1.08*** | -0.92*** | 12.25*** |
| Side effects | No** | 0.36*** | 0.63*** | 0.10** | 2.43** |
|  | Interactions with other medications | -0.11*** | 0.25*** | 0.01 | 0.38 |
|  | Mild | -0.02 | 0.15*** | -0.01 | 3.18*** |
|  | Rare chance of kidney problems* | -0.30*** | 0.51*** | -0.08* | 1.38 |
|  | Mild pain at injection | 0.07*** | 0.24*** | -0.02 | 3.71*** |
| Visit frequency | Every 2 months*** | -0.13*** | 0.22*** | -0.19*** | 3.78*** |
|  | 3 months | -0.02* | 0.02 | 0.01 | 2.00** |
|  | 6 months | 0.06*** | 0.21*** | 0.07 | 8.38*** |
|  | Once a year** | 0.09*** | 0.02 | 0.11** | 2.08** |
| Extra services | STI testing*** | 0.22*** | 0.36*** | 0.12*** | 0.73 |
|  | Hormones prescribed | NA | NA | 0.01 | 0.19 |
|  | Mental health counselling | -0.13*** | 0.36*** | -0.01 | 0.92 |
|  | None*** | -0.09*** | 0.06 | -0.12*** | 1.00 |
| Neither |  | -2.19*** | 4.42*** | -1.75*** | 22.05*** |

*significant at P<0.10, **significant at P<0.05, ***significant at P<0.01

GP=general practice, MSM= Men who have sex with men, NA=Not applicable, TGW= Transgender women, STI=sexually transmitted infection

**Table S4. PrEP preference between MSM at substantial- vs low risk of HIV infection**

| **Level** | **Substantial risk** | **Low risk** |
| --- | --- | --- |
| **Type of PrEP** |  |  |
| Daily Oral*** | 0.12 | 0.00 |
| On-demand*** | -0.13 | 0.20 |
| Injectable | 0.03 | 0.08 |
| Long-acting oral** | 0.07 | 0.23 |
| Implant** | -0.09 | -0.51 |
| **Location** |  |  |
| Hospital | -0.01 | -0.06 |
| STI clinic | -0.01 | -0.08 |
| Private community clinic (incl. GP) | -0.02 | 0.01 |
| Community clinic run by MSM | 0.02 | 0.14 |
| Telehealth | 0.01 | -0.03 |
| Pharmacy | 0.01 | 0.02 |
| **Cost** |  |  |
| Free*** | 0.16 | 1.21 |
| Low** | 0.07 | 0.24 |
| High*** | -0.09 | -0.33 |
| Very high*** | -0.14 | -1.12 |
| **Side effects** |  |  |
| No* | -0.05 | 0.34 |
| Interactions with other medications*** | -0.08 | -0.12 |
| Mild | -0.04 | -0.02 |
| Rare chance of kidney problems*** | 0.09 | -0.28 |
| Mild pain at injection | 0.08 | 0.08 |
| **Visit frequency** |  |  |
| Every 2 months** | -0.06 | -0.18 |
| 3 months | 0.04 | -0.02 |
| 6 months* | 0.05 | 0.07 |
| Once a year*** | -0.09 | 0.07 |
| **Extra service** |  |  |
| STI testing | 0.01 | 0.22 |
| Mental health counselling | 0.01 | -0.12 |
| None | -0.02 | -0.10 |

*significant at P<0.10, **significant at P<0.05, ***significant at P<0.01

GP=general practice, MSM=men who have sex with other men, STI=sexually transmitted infection

**Table S5. PrEP preference between MSM who were experienced- vs naive-PrEP users**

| **Level** | **Experienced PrEP users** | **Naive-PrEP users** |
| --- | --- | --- |
| **Type of PrEP** |  |  |
| Daily Oral ** | 0.07 | -0.08 |
| On-demand*** | -0.02 | 0.33 |
| Injectable*** | 0.22 | 0.01 |
| Long-acting oral | 0.22 | 0.22 |
| Implant | -0.49 | -0.48 |
| **Service location** |  |  |
| Hospital** | -0.01 | -0.09 |
| STI clinic | -0.10 | -0.08 |
| Private community clinic (incl. GP) | 0.01 | 0.02 |
| Community clinic run by MSM* | 0.19 | 0.13 |
| Telehealth** | -0.08 | 0.00 |
| Pharmacy | -0.01 | 0.02 |
| **Cost** |  |  |
| Free*** | 1.16 | 1.24 |
| Low | 0.25 | 0.21 |
| High | -0.32 | -0.33 |
| Very high | -1.09 | -1.12 |
| **Side effects** |  |  |
| No*** | 0.31 | 0.40 |
| Interactions with other medications*** | -0.17 | -0.08 |
| Mild | -0.01 | -0.01 |
| Rare chance of kidney problems*** | -0.22 | -0.38 |
| Mild pain at injection | 0.09 | 0.07 |
| **Visit frequency** |  |  |
| Every 2 months*** | -0.17 | -0.11 |
| 3 months*** | 0.02 | -0.06 |
| 6 months | 0.05 | 0.06 |
| Once a year | 0.09 | 0.11 |
| **Extra service** |  |  |
| STI testing*** | 0.23 | 0.21 |
| Mental health counselling | -0.12 | -0.12 |
| None | -0.11 | -0.09 |

*significant at P<0.10, **significant at P<0.05, ***significant at P<0.01

GP=general practice, MSM=men who have sex with other men, STI=sexually transmitted infection

**Table S6.** **Heterogeneity preference between TGW at substantial- vs low-risk of HIV infection**

| **Level** | **Substantial risk** | **Low risk** |
| --- | --- | --- |
| **Type of PrEP** |  |  |
| Daily Oral | -0.11 | -0.07 |
| On-demand | 0.05 | 0.18 |
| Injectable | 0.17 | 0.08 |
| Long-acting oral | 0.10 | 0.06 |
| Implant | -0.21 | -0.25 |
| **Service location** |  |  |
| Hospital | -0.14 | -0.12 |
| STI clinic | -0.03 | 0.22 |
| Private community clinic (incl. GP)** | -0.01 | -0.40 |
| Community clinic run by TGW | 0.21 | 0.22 |
| Telehealth* | 0.09 | -0.21 |
| Pharmacy*** | -0.12 | 0.29 |
| **Cost** |  |  |
| Free | 1.16 | 0.98 |
| Low | 0.11 | 0.12 |
| High | -0.32 | -0.36 |
| Very high | -0.95 | -0.74 |
| **Side effects** |  |  |
| No | 0.08 | -0.05 |
| Interactions with other medications | 0.03 | -0.06 |
| Mild | 0.03 | -0.06 |
| Rare chance of kidney problems | -0.08 | -0.13 |
| Mild pain at injection | -0.06 | 0.30 |
| **Visit frequency** |  |  |
| Every 2 months | -0.38 | -0.32 |
| 3 months | -0.03 | 0.12 |
| 6 months | 0.02 | 0.22 |
| Once a year | 0.14 | -0.02 |
| **Extra service** |  |  |
| STI testing* | 0.16 | -0.05 |
| Hormone prescribed*** | -0.04 | 0.28 |
| Mental health counselling | 0.00 | -0.04 |
| None | -0.12 | -0.19 |

*significant at P<0.10, **significant at P<0.05, ***significant at P<0.01

GP=general practice, SD=Standard Deviation, STI=sexually transmitted infection, TGW=Transgender women

**Table S7.** **Heterogeneity preference between TGW who were experienced- vs naive-PrEP users**

| **Level** | **Experienced PrEP users** | **Naive-PrEP users** |
| --- | --- | --- |
| **Type of PrEP** |  |  |
| Daily Oral | 0.03 | -0.14 |
| On-demand | -0.01 | 0.09 |
| Injectable | 0.24 | 0.15 |
| Long-acting oral** | -0.05 | 0.09 |
| Implant | -0.21 | -0.19 |
| **Service location** |  |  |
| Hospital | -0.09 | -0.27 |
| STI clinic | -0.04 | 0.10 |
| Private community clinic (incl. GP) | 0.01 | -0.09 |
| Community clinic run by TGW | 0.23 | 0.17 |
| Telehealth | -0.05 | 0.06 |
| Pharmacy | -0.06 | 0.03 |
| **Cost** |  |  |
| Free | 0.87 | 0.77 |
| Low | 0.12 | 0.13 |
| High* | -0.26 | -0.19 |
| Very high | -0.73 | -0.71 |
| **Side effects** |  |  |
| No | 0.06 | 0.10 |
| Interactions with other medications | 0.00 | -0.03 |
| Mild | 0.04 | 0.10 |
| Rare chance of kidney problems** | 0.04 | -0.15 |
| Mild pain at injection | -0.14 | -0.02 |
| **Visit frequency** |  |  |
| Every 2 months | -0.08 | -0.02 |
| 3 months | 0.01 | 0.01 |
| 6 months | 0.10 | -0.03 |
| Once a year | 0.04 | 0.04 |
| **Extra service** |  |  |
| STI testing | 0.02 | 0.03 |
| Hormone prescribed | -0.01 | 0.04 |
| Mental health counselling | -0.01 | -0.07 |
| None | -0.08 | -0.06 |

*significant at P<0.10, **significant at P<0.05, ***significant at P<0.01

GP=general practice, STI=sexually transmitted infection, TGW=Transgender women

**Figure S1. Relative importance of attributes for PrEP among men who have sex with men in 15 Asian countries and Australia (N=21,943)**

**Figure S2. Preference for PrEP among transgender women in 11 Asian countries (N=1,522)**

*significant at P<0.10, **significant at P<0.05, ***significant at P<0.01

GP=general practice, STI=sexually transmitted infection, TGW=transgender women
